# Heritable Genome Editing in a Global Context: *National and International Policy Challenges*

**DOI:** 10.1002/hast.1006

**Published:** 2019-07-03

**Authors:** Achim Rosemann, Adam Balen, Brigitte Nerlich, Christine Hauskeller, Margaret Sleeboom‐Faulkner, Sarah Hartley, Xinqing Zhang, Nick Lee

## Abstract

*A central problem for the international governance of heritable germline gene editing is that there are important differences in attitudes and values as well as ethical and health care considerations around the world. These differences are reflected in a complicated and diverse regulatory landscape. Several publications have discussed whether reproductive uses would be legally permissible in individual countries and whether clinical applications could emerge in the context of regulatory gaps and gray areas. Systematic comparative studies that explore issues related to the governance of this technology from different national and international perspectives are needed to address the lack of knowledge in this area. In this research report, we contribute to filling this gap by presenting views of stakeholders in the United Kingdom on challenges to the governance of heritable genome editing. We present findings from a multistakeholder study conducted in the United Kingdom between October 2016 and January 2018 and funded by the Wellcome Trust. This research included interviews, literature analysis, and a workshop. We involved leading U.K. scientists, in vitro fertilization clinicians, and representatives from regulatory bodies, patient organizations, and other civil societal organizations, as well as fertility companies. Part one of this article explores stakeholder perceptions of possible global developments in heritable genome editing and associated risks and governance challenges. Part two presents a range of policy options that were generated during the workshop in relation to the challenges discussed in part one*.

Commentators agree that heritable human genome editing poses a global governance challenge. A central problem is that governance is likely to differ around the world. What are the possible implications for patients and providers, and what are the possible policy responses? The findings of a multistakeholder study in the United Kingdom demonstrate the need for early and broad public deliberation.

## Article

In July 2018, the Nuffield Council on Bioethics in the United Kingdom published a report that concluded that the “use of heritable genome editing to influence the characteristics of future generations could be ethically acceptable in some circumstances,” as long as specific preconditions are met.^1^ The report followed a 2017 U.S. National Academies of the Sciences, Engineering, and Medicine (NASEM) publication that recommended permitting clinical trials for the prevention of severe monogenetic disorders, as long as these are conducted within a robust and effective regulatory framework.^2^ These reports and a range of other publications^3^ have also demanded that clinical research and applications should be preceded and accompanied by “extensive and inclusive public participation,”^4^ so as to collectively consider the possible uses, limits, and risks of gene editing technologies. A central concern of many of these studies has been to anticipate and reflect on the short‐ and long‐term societal implications of heritable genome editing, not only for people who are born as a result of genome editing, but also for current and future societies at large. Ethical issues raised in the publications include, among others, the significance of solidarity, social justice, and responsible innovation to prevent adverse medical effects, discrimination, disadvantages, and societal divisions.^5^

Then, on November 25, 2018, just days before the start of the Second International Summit on Human Genome Editing, in Hong Kong, news broke that a woman had given birth to two babies whose genomes had been edited by a research team in China.^6^ This use of genome editing was widely condemned as irresponsible and as failing to conform with international norms.^7^ It also had a significant impact on regulatory debates. The organizing committee of the Hong Kong summit declared, for example, the need for “a rigorous, responsible translational pathway toward” “clinical trials of germline editing” that establishes international “standards for preclinical evidence and accuracy of gene modification, assessment of competency for practitioners of clinical trials, [and] enforceable standards of professional behavior.”^8^ This statement was criticized, however, by both scientists and social scientists because it deemphasized the commitment to a “broad societal consensus” that was evident in the NASEM and Nuffield Council reports and in other publications.^9^ A group of scientists and ethicists working with the geneticist Eric Lander, for instance, now demanded the adoption of a temporary global moratorium on all clinical uses of human germline editing, arguing that the decision to move toward clinical applications should not be made by the scientific or medical community, but by societies as a whole.^10^ At the same time, the World Health Organization responded to the news of the genetically modified babies in China by setting up a new advisory committee to develop global standards for the governance and oversight of human genome editing, aiming to work toward a strong international governance framework.^11^ In a first step, this panel suggested the creation of a global registry of all human gene editing research, which would allow oversight and transparent access to the details of current and future studies by interested parties.^12^

A central problem for the international governance of germline gene editing is that there are important differences in attitudes and values as well as ethical and health care considerations around the world. These differences are reflected in a complicated and diverse regulatory landscape. Several publications have sought to develop a broad, comparative understanding of existing regulatory conditions in different countries^13^ and to sample international legal and regulatory frameworks that may influence the governance of clinical applications in this field.^14^ Other articles have explored the social and ethical dimensions of this technology in relation to a variety of topics, such as the interests and rights of people with disabilities;^15^ reproductive autonomy;^16^ the history of eugenics and racism;^17^ the gendered, ethnic, and socioeconomic dimensions of genome editing;^18^ the consequences of gene editing for future generations;^19^ and challenges related to informed consent.^20^ Other work has compared existing regulatory frameworks for basic and preclinical research as well as potential reproductive applications of human genome editing.^21^ A central point of discussion in these studies was whether reproductive uses would be legally permissible in individual countries and whether clinical applications could emerge in the context of regulatory gaps and gray areas.^22^

However, a systematic investigation of the possible effects of international regulatory differences, and of the challenges likely to arise for national governments once clinical applications in this field become available at a global scale, has not yet been conducted. Neither the recent Nuffield Council report, nor the NASEM report, nor other previous studies have systematically addressed this issue. This is an important shortcoming. The incident in China suggests that different forms of regulatory oversight as well as differences in social environments, clinical cultures, and patient needs will probably result in additional premature and irresponsible applications of heritable genome editing, which could expose prospective parents and their embryos, fetuses, and of course, children and their descendants to substantial risks.

Systematic comparative studies that explore issues related to the governance of this technology from different national and international perspectives are needed to address the lack of knowledge in this area. In this research report, we contribute to filling this gap by presenting views of stakeholders in the United Kingdom on challenges to the governance of heritable genome editing. We present findings from a multistakeholder study conducted in the United Kingdom between October 2016 and January 2018 and funded by the Wellcome Trust. This research included interviews, literature analysis, and a workshop. We involved leading U.K. scientists, in vitro fertilization (IVF) clinicians, and representatives from regulatory bodies, patient organizations, and other civil societal organizations, as well as fertility companies.

Part one of this article explores stakeholder perceptions of possible global developments in heritable genome editing and associated risks and governance challenges with respect to two overarching themes. The first theme is issues related to the situation and well‐being of fertility patients and babies whose genomes are edited. In considering these issues, we focus on the potential challenges of germline gene therapy tourism and the emergence of commercial clinics that are likely to offer potential patients germline editing of embryos for nontherapeutic purposes. Illegal and rogue clinics will operate outside of systematic clinical research protocols and oversight, prior to reliable clinical studies for the interventions they offer.^23^ Yet, as in other fields of medicine, some commercial clinics may claim to provide clinical applications as part of a research framework. Many clinics offer treatments in a “gray zone” between the two poles of “rigorous science” and “quackery.”^24^ This can make it difficult to draw a clear line between rogue clinical applications and legitimate clinical research.

**Methodology**

We designed our investigation of stakeholders’ views of the challenges and options for the governance of heritable genome editing with the methodological approach known as forward engagement, which aims to identify challenges that arise from new technologies as well as possibilities for adaptation and governance as far in advance as possible.^1^ Our aim was to initiate a systematic thought process among multiple stakeholders in the United Kingdom about long‐range issues in the governance of human genome editing and to help inform collective responses and policy responses at an early stage.^2^ To achieve this aim, we have combined multistakeholder deliberation, semistandardized interviews, video interviews, and documentary research.

The project consisted of two phases. Phase one involved eighteen semistandardized in‐depth interviews with U.K. stakeholders with varied professional backgrounds (as described in this article's introduction) as well as documentary research (policy reports, public commentary, and relevant academic publications).

Phase two consisted of the organization of a one‐day multistakeholder workshop that was held in June 2017 in London. The workshop methodology involved focus group discussions, plenary discussions, and short video interviews. Hypothetical case scenarios were used as a focus device to stimulate discussions of likely challenges and reflection on possible responses. The case studies were based on the phase‐one interviews and complementary insights from newspaper and media coverage. Three potential scenarios that are likely to occur in the next ten to thirty years were discussed: clinicians from countries in which heritable gene editing is prohibited collaborate with IVF clinics in more leniently regulated countries to achieve first‐in‐human applications in a legal gray area, transnational germline gene therapy tourism emerges, and commerce‐driven (and possibly fraudulent) applications of “genetic enhancement” surface. Parallel to the workshop, six 10‐ to 15‐minute video interviews were conducted. All data presented in this article have been anonymized to protect the interests of project participants and the organizations they represent.

The stakeholder‐participants in this research were based in the United Kingdom, and our findings have been shaped by their perspectives. Stakeholders from other countries may express other views or have different priorities. However, all the possible clinical developments discussed in this report concern global challenges and risks that are likely to affect humanity in general. The ideas and options presented here thus contribute to the ongoing international dialogue and inform academic and policy debates on heritable genome editing at national and international levels.

1

L. S.
Fuerth
 and 
E. M.
Faber
, Anticipatory Governance: Practical Upgrades (Washington, DC: National Defense University Press, 2012), http://www.dtic.mil/dtic/tr/fulltext/u2/a585519.pdf.2Ibid.

The second theme is issues for researchers, fertility clinics, and corporations that aim to develop, apply, and commercialize this technology in the context of international research or commercial partnerships. These partnerships can be built around the coorganization of clinical research studies, or they can target the provision of unproven, or nonsystematically evidenced, clinical interventions on a commercial basis in permissive locations.

In the second part of the article, we present a range of policy options that were generated during the workshop in relation to the challenges discussed in part one. While the options represent the views of stakeholders in the United Kingdom, they address issues and possible clinical (and commercial) developments of international relevance. The surfacing of premature, irresponsible, or rogue applications; the potential emergence of transnational germline therapy tourism; and the possibility of genetic enhancement are shared, global challenges. For this reason, the policy options put forward in this report have the potential to contribute to international dialogue and inform the development of collective responses to heritable genome editing. Before getting to these empirical findings, we briefly discuss possible applications of heritable genome editing technology and provide a more detailed overview of the study's methodology.

## Heritable Genome Editing: From Research to Applications

The bioethicist and policy analyst Tetsuya Ishii has noted three objectives for which heritable gene editing is expected to be used: the prevention of monogenic conditions (such as Huntington disease, Tay‐Sachs disease, and cystic fibrosis), the maximization of reproductive choices during in vitro fertilization (such as the prevention of baldness and the choice of eye, hair, and skin color), and genetic enhancement (for example, for height, muscularity, and learning and memory—although these are not all attributable solely to genetics).^25^ Current recommendations to permit clinical trials with genetically modified gametes, zygotes, or embryos relate exclusively to the prevention of severe genetic disorders. However, as Ishii has pointed out, while certain increased reproductive choices and genetic enhancement are seen by many as problematic, in a diverse regulatory environment, the emergence of these applications in artificial reproductive technology centers seem likely in the mid‐ to long term.^26^

Others have made similar assessments. The NASEM Committee on Human Genome Editing warned, for instance, that “regulatory havens” could emerge that would tempt providers or consumers to travel to jurisdictions with more lenient or nonexistent regulations to undergo procedures that are prohibited in other countries.^27^ Developments in the field of regenerative medicine have shown, for instance, that in many countries, for‐profit applications emerge long before reliable regulatory controls are in place, often in a legal gray area and with problematic consequences.^28^ Considering the potential implications of heritable gene editing for human societies and the fact that germline engineering will be difficult to control at a global level,^29^ an exploration of possible developments, challenges, and risks in this field is important.

A more general problem with the clinical translation of heritable genome editing technology is that it is difficult to draw a clear line between clinical applications and clinical research. Some clinics, as we have stated above, may offer clinical applications of heritable genome editing on a commercial basis outside of rigorous (internationally accepted) research protocols and oversight, but they still may be involved in preclinical research or may implement follow‐up procedures that monitor the genetic or functional effects of the intervention during pregnancy, after birth, or during childhood and adulthood.

Another problem is that the established clinical translation pathway for new therapies—namely, multistage controlled trial system to determine the safety and efficacy of a tested treatment—is not available for heritable genome editing. Germline therapy cannot be part of a controlled study design because there are no controls (except other “unmodified” individual embryos and human beings) for comparison. In addition, exploratory (phase zero or phase one) trials that use only very small doses of a new drug in a small number of patients to establish its safety cannot be conducted for germline therapy. In standard first‐in‐human trials, an administered drug can be withdrawn instantaneously after the emergence of adverse effects, and, if necessary, treatments can be provided to counter adverse effects. For germline gene therapy, these options do not exist. Once a genetically modified embryo has been implanted into the uterus, the intervention cannot be reversed. If prenatal diagnosis reveals a defect caused by the gene therapy that will seriously affect fetal development and the future life of a person after birth, the primary options to prevent the negative effects of the treatment are through abortion or, where legal, postnatal euthanasia. If the fetus develops into a baby who survives, the person will have to live with the consequences of the intervention for the rest of her or his life and might transmit the effects to subsequent generations. In consequence, every reproductive intervention, even a first‐in‐human application that is part of a systematic research protocol, is an immediate full clinical application whose effects cannot be reversed.

These considerations do not rule out the possibility of making and maintaining a clear distinction between systematic fully monitored and recorded research‐based applications and other—ethically more problematic—forms of experimental for‐profit applications. Nevertheless, the fuzzy boundaries between clinical research and clinical applications will have important regulatory implications if and when a translational pathway and oversight for heritable genome editing are developed.

## Challenges for Fertility Patients and Babies

During the first phase of the project, two central themes emerged that were examined further in the context of the multistakeholder workshop: the rise of reproductive tourism and the expansion of rogue IVF clinics that offer gene editing for nontherapeutic purposes.^30^

***Germline therapy tourism*.** Reproductive tourism is a form of medical tourism in which fertility patients travel to other countries to “receive a specific treatment or to exercise personal reproductive choice.”^31^ These reproductive travels are frequently based on the legal prohibition in some countries of specific technologies that, in some other countries, are legal or are tolerated in a gray zone. The legal analyst Glenn Cohen has called these forms of medical travel “circumvention tourism” because they circumvent domestic prohibitions on accessing specific medical services.^32^ Surrogacy is a good example. As the South African sociologist Amrita Pande has shown, fertility patients from high‐income countries in which surrogacy is banned travel to India and other societies where surrogacy is permitted and surrogacy mothers can be hired for a low payment.^33^ Many of our research participants expected that circumvention tourism could also occur with heritable gene editing, at least as long as these treatments are not accessible in the United Kingdom. Participants assumed that IVF clinics in countries without regulatory frameworks or with permissive or flexibly enforced ones were likely to provide germline gene therapy for monogenic disorders much earlier than clinics in countries with more restrictive regulatory controls. Many participants saw this situation as both a threat to the well‐being of parents and children and a challenge to the future development of this field.

While most participants said they understood the motivation of prospective parents to seek germline gene therapy for children who would otherwise suffer from severe genetic disorders, they also expressed a range of concerns, some of which were summarized by a senior IVF clinician in our workshop:

Parents, [have] the desire to seek the best for their future children. So we understand why, if the treatment was not permissible in the U.K., parents may wish to go overseas. However, we felt strongly that any such treatment needs to be part of a continuum of appropriate preclinical and then clinical studies, transparent and open with proper ethical review and follow‐up. And, of course, we were concerned about … a child being born [and coming] back to the U.K., needing to be looked after and followed‐up by the NHS [National Health Service], and the potential implications [of this].

The main concern here is that, in some countries that lack or have lenient regulatory conditions, early‐stage reproductive applications could, and likely will, be provided outside of a systematic research framework and also independent of the review and approval mechanisms of regulatory agencies. Most interviewees stated that this would be problematic for the responsible development of heritable genome editing technology as a whole because it would prevent the generation and publication of reliable data, including of negative results.

One issue highlighted in interviews and workshop discussions was that the early‐stage provision of germline therapies in some clinics was likely to be driven primarily by financial motives or professional vanity, instead of a strong clinical justification and reliable preclinical data. As several interviewees mentioned, in a global environment where the generation of profits is often more important than scientific integrity, clinical applications may be offered prematurely and irresponsibly. As a result, fertility patients, their embryos, children, and subsequent generations could be exposed to significant unjustified psychological and health risks. Potential adverse effects or problems, such as increased miscarriage rates, are likely to be kept secret and not to be shared with the scientific community. The criteria on which institutional or ethics review boards have based their assessments and approvals of reproductive applications are also likely to remain unclear.

Participants also expressed concern about the risks of missing long‐term follow‐up monitoring in overseas IVF clinics, especially if patients travel to these clinics from abroad. To establish a reliable evidence base, the U.S. National Academies of the Sciences, Engineering, and Medicine^34^ and other researchers^35^ have recommended long‐term, possibly multigenerational, follow‐up of individuals who have received heritable gene editing.

Still other concerns relate to legal responsibilities: Who is liable for reproductive applications in other countries if something goes wrong? Can patients take legal action against an overseas clinic if their child suffers from (potentially irreversible) adverse effects? Will the NHS be ready to pay for subsequent treatments and care arrangements? As research on other areas of medical tourism has shown, medical tourists paying out of pocket often face the problem of legal liability. Patients face a lower likelihood and degree of recovery for adverse effects or injuries sustained as a result of medical tourism.^36^ Moreover, such adverse effects may require follow‐up treatment in patients’ home countries, which can cause additional costs for national health systems.

***“Rogue” IVF clinics and germline therapy for nontherapeutic purposes*.** Reproductive tourism sometimes involves the use of rogue clinics. People travel, for example, to China to use clinics offering surrogacy and sex selection, practices that were outlawed there in 2001. Despite the government's repeated efforts to close down this market and despite the risk of punishment, clinics using artificial reproductive technology (ART) have been incentivized by a steady demand for these services.^37^ Illegal or gray‐area applications in stem cell medicine are another example. As reported by Dominique McMahon,^38^ a global industry of rogue stem cell clinics has emerged since the mid‐2000s. Hundreds of clinics in numerous countries, including the United States and other high‐income countries, offer unauthorized stem cell interventions whose safety and effectiveness have not been systemically proven.^39^

Many of our interviewees expressed concern that similar developments might occur in regard to heritable gene editing. The surfacing of rogue clinics was seen as particularly likely in countries with no regulation or lenient regulatory infrastructures. A senior researcher stated that excitement about heritable genome editing could lead to hype and fuel the provision of heritable genome editing in illegal or gray‐area ART clinics. He and other respondents considered it very likely that unscrupulous, misguided, or inexperienced clinicians will offer germline therapy to patients who desire a child without a genetic disease. Similar concerns have also been expressed by the bioethicist and legal scholar Alta Charo, who also has warned of the emergence of germline therapy tourism.^40^ According to Charo, medical and reproductive tourism is not necessarily a bad thing, but it should take place only after the development of safe and effective interventions and should not precede or be a part of the clinical research and development process.

Interviewees expected that these clinics would initially focus solely on the correction of monogenic medical conditions, but that, over time, once germline therapy for single gene disorders was more widely available and accepted, clinics would also provide treatments for polygenic conditions and nontherapeutic purposes, including forms of genetic enhancement. CRISPR coinventor Jennifer Doudna conveyed similar worries during her 2017 visit at the Royal Society: “I certainly hear about work … that is aimed at helping in vitro fertilization clinics to apply [germline gene editing] for correcting genetic diseases, or for making other kinds of genetic changes. And also to commercialize that. And that is … where I feel more uncomfortable. I certainly feel more uncomfortable with a company trying to make money, telling people …. “Hey, you can have a better child … when you do this …”^41^

Project participants defined “rogue” IVF clinics in various ways. The most important criteria used to differentiate these clinics from providers of more “legitimate” clinical services were that they were likely to:

- provide insufficient information about the kinds of methods and protocols they use;
- work with false claims and misleading advertisement strategies;
- provide clinical services that are based on insufficient forms of preclinical or clinical evidence;
- offer nontherapeutic or enhancement applications in a legal gray area or even in the context of legal prohibition;
- sell potential “snake oil” interventions, which would not involve gene editing at all despite being sold and advertised as such interventions; and
- expose IVF patients and their offspring to uncertain and unjustifiable risks.

Participants identified four key policy challenges related to illegal or rogue IVF clinics offering heritable gene editing to patients. The first is to make sure that germline gene editing is provided only in a well‐regulated, controlled environment. This would include preventing clinical entrepreneurs from trying to exploit the desire of prospective parents to have a healthy baby with specific physical, mental, or cognitive characteristics, at least as long as the clinical utility of these interventions was not reliably established and a public consensus on the desirability of these applications was not yet achieved.

A second challenge is to protect patients from deceptive marketing and advertising, including that without sufficient information about possible adverse effects and risks. Public education of risk groups and caregivers was seen as a means to achieve this goal, to inform people of the most recent stage in the technique's developments, about potential individual and societal consequences, and about possible adverse effects and risks. Such education could be developed and offered by patient organizations, health service providers, and regulatory bodies.

Participants saw the emergence of enhancement‐oriented forms of germline therapy as another challenge. Numerous interviewees expected that, in the mid‐ to long term, clinical applications intended to achieve some form of genetic enhancement would be offered to patients. Because these interventions would inevitably involve the modification of large numbers of gene locations, the risk of adverse effects was expected to be considerably higher than for the treatment of monogenic disorders. Most respondents argued in favor of a prohibition against enhancement applications but recognized that, at a global level, such a ban would be difficult to achieve.

Finally, participants identified the need to avoid the provision of fraudulent interventions, that is, clinical applications that claim to involve germline therapy when in fact they do not. The likelihood of such deceitful interventions was seen to be particularly high with regard to nontherapeutic or “enhancement” applications—because the actual efficacy and effects of these “treatments” will be extremely difficult to verify. Interviewees pointed out the following:

- “In principle—as happened with for‐profit stem cell interventions—clinics or corporations can work with entirely fraudulent claims” (IVF Clinician 1).
- “One would never know how well it would have worked” (Senior Researcher).
- “There will be a lack of evidence for these interventions, but private clinics and corporations are likely to do it nonetheless” (IVF Clinician 2).

These concerns closely echo Ishii's estimation that nontherapeutic and enhancement applications are probably inevitable at a global level and are only a matter of time.^42^ They also resonate with Harald König's assessment that there is an “illusion of control” in many of the current policy debates on germline engineering.^43^ He has pointed out that a central assumption in current discussions on the governance of heritable genome editing is the notion that “technologies can be prohibited … until they are ‘safe enough’—and, moreover, that this can be done globally.”^44^ According to König, it is an illusion that such a universal level of control can be achieved, partly because current regulations for germline genome editing vary considerably across the globe, partly because there are different ethical and moral ideas on human genome editing, and partly because of the economic incentives that drive innovation processes in this field.^45^

## Challenges Related to Researchers, Fertility Clinics, and Corporations

A second set of policy challenges that the project examined related to the situation and activities of researchers, fertility clinics, and corporations that are likely to develop, apply, and commercialize this technology over the course of the next years and decades. Throughout the interviews and workshop, our aim was to identify obstacles to the realization of responsible forms of transnational research and corporate practices in an environment that is characterized by significant differences in research cultures, regulatory structures, and business and commercialization practices.

***U.K. researchers and corporations operating abroad to avoid regulatory restrictions*.** A widespread concern among participants was that U.K. researchers, clinicians, and fertility companies would seek opportunities to provide and commercialize heritable genome editing applications in more permissive countries, especially as long as the technology was not permitted in the United Kingdom. An example that came up repeatedly in interviews and group discussions was the first application of mitochondrial gene transfer in Mexico in 2015, a case in which U.S. clinicians traveled to Mexico to create a “three‐parent” embryo that then developed into a healthy baby, despite the fact that the technology was not approved in the United States.^46^ Participants expected that a similar development could occur with regard to heritable genome editing. In open discussions during the workshop, there was unanimous consensus that if these clinical applications were not part of systematic and formally approved clinical studies, this was bad practice that should be addressed and prevented by U.K. government bodies, research councils, and scientific and medical organizations.

In interviews, a widely expressed concern was that the operation of U.K. researchers and corporations in more permissively regulated countries could harm the scientific status of the United Kingdom and prevent progress for the clinical validation of heritable genome editing through a focus on profit opportunities rather than robust data. However, some interviewees supported the idea that U.K. researchers and fertility companies could provide heritable genome editing treatments in more leniently regulated countries if these collaborations would enable U.K. patients to access potentially beneficial interventions for severe genetic disorders that are not yet available in the United Kingdom. This view was particularly notable among representatives of patient organizations. These spokespersons argued that preventing patients from seeking potentially beneficial treatments abroad was a violation of patients’ rights and ignored patients’ suffering. One of the representatives stated that acknowledging reproductive freedom and parental autonomy is especially important in the context of the burden and inevitability of serious genetic diseases and of the instinctive and intuitive desire of parents to have an unaffected child. The spokesperson of another patient organization stressed that the knowledge that a potential treatment was available, albeit in a different country with less rigorous regulations, instills a psychological motivation to use this treatment. That is why, from his view, inaction by the U.K. government to permit heritable genome editing for monogenic disorders would ultimately encourage patients to travel to other countries.

***Involvement of U.K. researchers in overseas heritable genome editing research trials*.** Another form of transnational interaction discussed during the project was the involvement of U.K. researchers in overseas clinical research studies that would involve heritable genome editing. While interviewees thought that, at present, any form of clinical research in this field was premature, there was a consensus that once the technology was proven to be safer and more reliable, participation in international clinical studies would be acceptable. There was also a widespread agreement that such interventions must be provided as part of a systematic, science‐driven clinical study format and that these studies should be formally approved by a government agency and conducted in line with international guidelines. As a set of minimum criteria, participants suggested the following benchmarks: treatment protocols are transparent, independent ethical review occurs, approval comes from a national‐level government agency, clinical applications are based on sufficient preclinical evidence on safety and efficacy, such applications follow from a convincing medical rationale that justifies the interventions, and clinical interventions are conducted in the context of an international dialogue and under systematic, independent peer review. Participants judged that, if these conditions were met, involvement of U.K. researchers in overseas heritable genome editing trials would be acceptable even if such trials were still prohibited in the United Kingdom.

## Policy Options

How should policy‐makers, professional bodies, and other stakeholders respond to the challenges we describe in this article to maximize patient safety and to enable responsible forms of clinical translation and ethically robust forms of international research and corporate collaborations? In the context of interviews and the workshop, project participants developed six broad policy options.

***Proactive regulation*.** The majority of participants argued in favor of proactive legislation that would enable clinical heritable genome editing research under carefully defined conditions. As the director of an IVF unit put it, the creation of a permissive but carefully regulated research environment for heritable genome editing in the United Kingdom would prevent reproductive tourism and allow initial clinical applications under a systematic research framework:

If [this technology] is found to be safe, and only if it is found to be safe, [we must] ensure that we have tight, permissive regulation enabling those parents who may want to go through these therapies for their future children to have the ability to have safe therapies in the U.K., rather than feeling the need to go overseas and to be potentially treated by rogue clinicians, rogue scientists, in rogue clinics, where we won't necessarily have insight into the failures, the mistakes, the problems that may occur. (Interviewee 14)

Several participants emphasized that the United Kingdom has a comprehensive and mature regulatory framework that governs ARTs, embryo research, and the creation and use of human embryos for research purposes and that this framework can, in principle, be extended to regulate first‐in‐human applications of heritable gene editing. While there was a shared understanding that it was now too early to change legislation and permit clinical applications, various participants thought it was crucial that the U.K. government start to anticipate and address some of the key concerns of heritable genome editing technology and to begin developing appropriate policy responses. A senior researcher, for instance, mentioned that the U.K. government needs to “think about what forms regulation would take to govern this area of potential clinical practice.” He also stated that “it is quite clear that we are not ready yet. There are still lots of issues that need to be sorted out. But it is important to have government … start thinking about this now, as an issue” (Interviewee 12). This view is also reflected in the statement of a senior IVF clinician: “This technology field is developing extremely quickly, and so we need to take stock of things, that it is properly regulated and that we have appropriately published preclinical studies before we bring this technology into clinical practice” (Interviewee 21).

While project participants did not have an answer to the question of when first‐in‐human applications should be allowed, they acknowledged that there is a fundamental tension between the development of a slow and careful regulatory framework in the United Kingdom and the pressures U.K. science is likely to experience from developments in other parts of the world. As the same senior IVF clinician put it,

At the moment, I think it is essential … that we move cautiously, that we move slowly, but at the same time, we have to recognize that there are scientists out there in the world who may be moving faster than we would like, and that brings the danger of people going overseas for treatments that are not regulated in the U.K. So if we accept that these sorts of therapies are going to come into clinical practice, we need to ensure that our regulation occurs in a timely manner. (Interviewee 21)

***Broad public engagement*.** A closely related policy option that project participants repeatedly emphasized was the initiation of far‐reaching public engagement exercises. A widespread view was that the United Kingdom is currently lacking in public and policy debates on reproductive gene editing and on the potential uses of heritable genome editing. Broad public engagement was seen as a central requirement to ensure that regulatory options and policies would correspond to the needs and perceptions of patients, laypeople, and society at large. A senior researcher summarized this view:

Accompanied with [a reflection on how clinical applications could be regulated] there [have] to be robust public engagement exercises, where you really get to not only give the information to the public but of course also get the feedback from the public of what they think might be useful, might be acceptable, and where are the limits. Because of course, there [have] to be limits applied to the use of this technology. There are potential uses which are beneficial, to avoid having serious genetic diseases. But there is a whole spectrum going towards more trivial applications, or even of enhancement, which would be really unhelpful and potentially lead to a public rejection of the whole notion. (Interviewee 11)

Several participants emphasized that, in the United Kingdom, a revision of the law that would enable clinical heritable genome editing applications could take place only if the wider public embraced this idea and if these applications lay within the limits of what society considers acceptable. In Great Britain, an effort to change the law would be initiated by the Department of Health. As part of this process, the DOH would first commission the Human Fertilisation and Embryology Authority to conduct public dialogue activities. The HFEA, in its function as a government regulatory body, is supposed to be neutral about any legislative change, and HFEA staff would not lobby government to change the law. However, following an order from the DOH, the HFEA would reach out into the public sphere and start a multistakeholder deliberation process. Based on the results from this process, the DOH would make a final decision. This was the procedure that led, for example, to the legislative change that permitted mitochondrial replacement therapy in the United Kingdom in 2016.^47^

However, for heritable genome editing, a different procedure might be used. The recent report from the Nuffield Council on Bioethics recommended that the coordination of societal debate on genome editing be done by an independent body or commission, and not a government body.^48^ Similar views were expressed during the workshop and interviews that inform this article. As a senior policy advisor pointed out, a robust public discussion should go way beyond the deliberation activities of the HFEA. Consultation of public opinions, according to this policy specialist, should be instigated by various parties, independent social scientists, and the HFEA, but also from within patient organizations, religious groups, and other social and civil societal organizations. “There is a need,” this individual asserted,

to have early upstream public engagement that … can feed into the policy‐making process…. I think it should be [initiated by] all sorts of different people. Researchers and [scientific] institutions have to be willing and open to talk about what their researchers are doing. Funders have to be open about it. It [this openness] has to be part of public discourses, you got to allow patient groups and consumer groups and all other people to have access to this information, when they go to these [deliberation] fora and talk about how they feel about this. (Interviewee 9)

Patient groups and other organizations are, of course, likely to use results from public deliberation to press for a more permissive legislation, at least if the majority of their members want this. The representatives of the two patient organizations that took part in our project stated, for example, that they would actively seek to lobby for regulatory change if their communities considered reproductive gene editing as a desirable option.

***International guidelines*.** Virtually all interviewees agreed that the development of an international consensus and international guidelines that define how heritable genome editing should be translated into clinical practice was essential. While participating stakeholders acknowledged that the development of international standards would not reach all clinics or researchers in this field, certainly not at a global level, many project participants supported the idea.

They saw international guidelines for the clinical translation of the technology as a way to promote unified technical and safety standards that could facilitate international knowledge exchange, dialogue, and collaborations, and as a basis to identify rogue clinics so as to warn patients of irresponsible or premature applications. However, a challenge for the development of international guidelines is that the technical procedures and effects of clinical heritable genome editing interventions are different for each disease. As the head of an IVF unit stated, the genetic manipulation for each genetic condition will be different, and there will be different risks. There thus needs, as was discussed in the workshop, to be a clearly defined strategy and pathway for each genetic problem. This need points to the potential limits of general international guidelines. It indicates that, beyond the development of a set of broad, overarching criteria such as transparent treatment protocols, reliable preclinical evidence, careful peer review, and approval by regulatory agencies, more detailed criteria and considerations will be required for specific genetic conditions so that the health and safety of newborns can be ensured.

Another problem participants mentioned is that international guidelines are not legally enforceable and that the implementation of these standards is likely to differ across the world. A widespread expectation was that individual clinics and researchers would try to circumvent these international norms, possibly at a larger scale. As one participant said,

History has shown that certain jurisdictions have been willing to tolerate, or perhaps turn a blind eye to irresponsible therapeutic applications, [more recently] particularly in relation to stem cell therapies, which have been offered without any evidence‐base for their human use…. I am more skeptical of international regulations and treaties and their effects, because … there are those who are willing to pursue commercial gain over the responsible practice of medicine, and there is a limit to what is going to effectively be done on a global scale to prevent this happening. (Interviewee 19)

While most participants acknowledged these challenges, they thought nevertheless that international guidelines would be an important and necessary regulatory instrument to help prevent misuse and to establish a solid evidence base.

***Scientific sanctions*.** Another policy option that project participants generated was scientific self‐governance and the enactment of scientific sanction. Although participants assumed that irresponsible clinical practices in the United Kingdom could be prevented (or, if necessary, addressed) by national law, the use of scientific sanctions was especially seen as a tool to discipline U.K. clinical researchers or corporations that would engage in irresponsible research or commercial activities overseas. Participating stakeholders were concerned that, by operating outside of the United Kingdom and in countries with permissive, ineffective, or not yet fully formed regulatory frameworks for heritable genome editing, these clinicians, researchers, or companies could effectively circumvent U.K. law. Most participants saw scientific sanctions as the most effective way to prevent problematic clinical commercial and research practices. A senior IVF clinician summarized this position:

I think what worries many of us is how this technology could be abused and misused and taken further than the desire to prevent debilitating diseases, more towards the slippery slope toward enhancing the human race in ways that are akin [to] the eugenics programs that we are all too aware of, from the last century. That is not a route that we want to go down. And therefore, I think that this technology needs to be very carefully considered and that we need not only to have international consensus but the ability to use sanctions against those who may misuse the technology. (Interviewee 21)

According to most participants, strong sanctions were in the interest of both patients and the scientific community. The following types of sanctions were mentioned: excluding researchers from professional societies and international bodies or committees, preventing researchers from accessing funding, preventing publications in top journals, placing notifications of fraudulent work in scientific journals and the media, and pressuring overseas clinics that collaborate with “irresponsible” U.K. researchers and corporations by clarifying that they operate outside of internationally acknowledged norms.

A limit to these sanctions is that they all apply to researchers, yet clinicians and entrepreneurs who are not part of the mainstream scientific community may not be effectively controlled by them. While the nature and implementation of these sanctions require further thought, interviewees thought that the enactment of sanctions should lie in the hands of a variety of stakeholders, such as professional organizations, funding bodies, review committees, and journals.

***Public and patient education*.** A fourth policy option addressed the question of how patients could be prevented from seeking access to risky and premature heritable genome editing applications overseas. Representatives of patient organizations and IVF clinicians concluded that alerting patients to up‐to‐date facts on the stages of clinical development, details on possible adverse effects and risks, and information about deceptive marketing and advertisement strategies would be the most promising strategy to influence patient behavior.

The dissemination of such information, as several participants pointed out, should involve multiple stakeholder organizations, such as the HFEA, the Medicines and Healthcare Products Regulatory Agency, the NHS, and other government bodies, scientific associations, and, of course, patient organizations. The representative of a patient organization mentioned that “disease societies … need to put [this information] prominently on the front page of their websites. If you are seeking a treatment for this and you contemplate going abroad, make sure that what is being offered is being done as part of a properly conducted trial for the following condition. And do not do it otherwise, because you are most likely not benefiting yourself” (Interviewee 14).

A different mechanism to create awareness among patients that participants suggested was to publish examples of irresponsible or fraudulent treatments and clinics in the media, including social media and websites of patient organizations and the NHS. As one participant said,

It is going to happen somewhere. It might happen because of the vanity of scientists or clinicians. It might happen because of the vanity or ego of prospective parents: “I slip you 100,000 dollars to do this.” Whether they [clinicians] keep it quiet or whether they publish it depends on how vain or ego driven they are…. So, make big examples of those [cases]. Publicize those examples, and hopefully, this will deter clinicians, bad clinicians, from offering these treatments, and hopefully, it will deter members of the public [from] seeking them out. (Interviewee 9)

***Regulation of advertisements*.** Another option that project participants identified was to look at, and possibly adjust, advertising legislation. To protect patients from false claims, careful consideration of acceptable forms of advertising for heritable genome editing therapies will become important, at least once first clinical applications become available. This might require the adjusting of advertising legislation so that it is compatible with the specific characteristics of heritable genome editing therapies and to create the legal basis for penalties, sanctions, or punishments and the introduction of a consumer‐focused complaints system.

A potential problem, especially in the context of transnational reproductive services, is differences in advertising legislation across jurisdictions. Moreover, the expansion of the Internet and social media have created new possibilities for the marketing of reproductive and therapeutic services. The ability of online advertising to reach worldwide audiences makes the enforcement of national laws more difficult, if not impossible.^49^ Due to the decentralized and borderless nature of the Internet and to more affordable international travel, national governments alone cannot solve the problem of irresponsible, transnational advertising practices.^50^

A key question discussed in the workshop was whether U.K. companies that provide heritable genome editing therapies abroad and that use false or misleading advertisements to attract customers in the global market could be prosecuted under the U.K. legal framework for advertising. A legal expert in international health law explained that this was conditional on where the company operates and where it is advertising. Much depends on the legal situation in the countries where the advertising is taking place and whether false advertising can be prosecuted under domestic law. In the United Kingdom, for example, the Advertising Standard Authority can take steps to enforce the removal or amendment of ads that breach the rules of Consumer Protection from Unfair Trading Regulations, a statutory instrument that was introduced in 2008. Failure to abide by the regulations can also result in fines and prosecution.^51^ In other words, at least in the United Kingdom, legal action has to come from within the country where companies operate and advertise their services.

There was widespread consensus that problematic advertisement strategies of U.K. companies abroad should be criticized from within the United Kingdom. Numerous interviewees thought that the U.K. government should take an active role in liaising with governments in countries where U.K. companies are likely to offer controversial heritable genome editing therapies. Another option discussed by participants was that the U.K. government should actively discourage companies from offering controversial clinical interventions overseas, for example, by imposing sanctions for U.K. professionals and companies planning to offer nonmedical or “enhancement” applications overseas, even if the applications are made available only to participants in research studies.

## Toward an Inclusive, Global Dialogue

The stakeholder views presented here provide insights into possible future developments, challenges, and risks when human germline gene editing is translated from the laboratory into clinical practice. Some of the stakeholder comments may make assumptions or provide interpretations that look unwarranted or unusual from other national or regional perspectives. However, at present there is no comparable work from other settings. Further, there is no global viewpoint available from which these assumptions, expectations, and interpretations could be judged. This matter of perspective points up the benefits of conducting this kind of research. It is exactly these expectations—well founded or not—that will inform policy development in the United Kingdom.

The findings demonstrate the complexity of the task to consider the prospects, risks, and the regulatory requirements and conditions within which this technique might be developed safely. Research participants suggested that there is an urgent need to engage with these challenges at an early stage of public deliberation and policy development. They have suggested six policy options that, if developed further, may have the potential to address and possibly prevent some of the challenges that this article identifies. At a more general level, it will be crucial to raise awareness of these issues in various contexts and to conduct further research to assess the impact of regulatory, social, scientific, and cultural variation in greater detail, with the aim to inform policy‐making as the technology develops and a consensus to translate it into clinical applications becomes more widespread.

Medium‐term, public engagement with multiple stakeholders at an international level will be important. This would allow the comparison of expectations and assumptions from different countries and would initiate a conversation on the development of potential future solutions to identified challenges.

## Acknowledgments

This work has benefitted from research support provided by the Wellcome Trust (204799/Z/16/Z). We would like to thank the participants of this research project and the two anonymous referees for their constructive comments.

## Privacy Statement

Due to ethical concerns, the research data supporting this publication are not publicly available.
